# Liver transcriptome analysis reveals extensive transcriptional plasticity during acclimation to low salinity in *Cynoglossus semilaevis*

**DOI:** 10.1186/s12864-018-4825-4

**Published:** 2018-06-18

**Authors:** Yufeng Si, Haishen Wen, Yun Li, Feng He, Jifang Li, Siping Li, Huiwen He

**Affiliations:** The Key Laboratory of Mariculture (Ocean University of China), Ministry of Education, Ocean University of China, Qingdao, People’s Republic of China

**Keywords:** Transcriptome, Osmoregulation, Salinity adaptation, Liver, *Cynoglossus semilaevis*, RNA-seq

## Abstract

**Background:**

Salinity is an important abiotic stress that influences the physiological and metabolic activity, reproduction, growth and development of marine fish. It has been suggested that half-smooth tongue sole (*Cynoglossus semilaevis*), a euryhaline fish species, uses a large amount of energy to maintain osmotic pressure balance when exposed to fluctuations in salinity. To delineate the molecular response of *C. semilaevis* to different levels of salinity, we performed RNA-seq analysis of the liver to identify the genes and molecular and biological processes involved in responding to salinity changes.

**Results:**

The present study yielded 330.4 million clean reads, of which 83.9% were successfully mapped to the reference genome of *C. semilaevis*. One hundred twenty-eight differentially expressed genes (DEGs), including 43 up-regulated genes and 85 down-regulated genes, were identified. These DEGs were highly represented in metabolic pathways, steroid biosynthesis, terpenoid backbone biosynthesis, butanoate metabolism, glycerolipid metabolism and the 2-oxocarboxylic acid metabolism pathway. In addition, genes involved in metabolism, osmoregulation and ion transport, signal transduction, immune response and stress response, and cytoskeleton remodeling were affected during acclimation to low salinity. Genes *acat2*, *fdps*, *hmgcr*, *hmgcs1*, *mvk*, *pmvk*, *ebp*, *lss*, *dhcr7*, and *dhcr24* were up-regulated and *abat*, *ddc*, *acy1* were down-regulated in metabolic pathways. Genes *aqp10* and *slc6a6* were down-regulated in osmoregulation and ion transport. Genes *abat*, *fdps*, *hmgcs1*, *mvk*, *pmvk and dhcr7* were first reported to be associated with salinity adaptation in teleosts.

**Conclusions:**

Our results revealed that metabolic pathways, especially lipid metabolism were important for salinity adaptation. The candidate genes identified from this study provide a basis for further studies to investigate the molecular mechanism of salinity adaptation and transcriptional plasticity in marine fish.

**Electronic supplementary material:**

The online version of this article (10.1186/s12864-018-4825-4) contains supplementary material, which is available to authorized users.

## Background

Salinity is an important environmental factor for marine fish aquaculture [1]. Fluctuations in salinity have a significant impact on fish reproduction, growth, development, and physiological and metabolic activities [2]. However, the molecular mechanism underlying salinity adaptation is not clear. Euryhaline fish species can adapt to a wide range of salinities and cope with both chronic and rapid osmotic stress [3], thus provide an excellent model to study osmoregulation during acclimation to various aquatic environments. The half-smooth tongue sole (*Cynoglossus semilaevis*) is a euryhaline fish species that can survive in a wide range of salinities range from 14 to 37 ppt (parts per thousand) [4]. The fish is mainly distributed in the East Asia and has emerged as an important commercial fish in aquaculture in China. The optimal salinity is 30 ppt in aquaculture [4, 5].

Osmoregulation is one of the most energetically costly metabolic activities in teleosts [6]. A large amount of energy is consumed by fish to maintain their osmotic homeostasis during acclimation to either freshwater or hyper-saline water [2, 7]. The liver, as the essential metabolic organ in fish, has been recognized as the major source of carbohydrate metabolites for osmoregulatory organs [2, 8]. In addition, the liver participates in certain important physiological processes such as antioxidant activity, glycogen synthesis and bile secretion in teleost fish. Long-term salinity stress inevitably leads to metabolic changes in the liver. However, compared with the widely studied osmoregulatory organs such as the gills, kidney and intestine, the liver response to osmotic stress is hardly known in teleosts [2, 9].

Previous studies on salinity stress response were mainly focused on the physiological and biochemical aspects [10], and molecular studies have been limited to the cloning and expression level detection of few osmoregulation genes [10–13]. Identification of the candidate genes involved in salinity adaptation is the first step in elucidating the molecular basis underlying osmoregulation [2]. Previous studies have identified several genes involved in the acute osmotic-stress response including Na^+^/K^+^-ATPase (*NKA*) [12, 13], vacuolar-type H^+^-ATPase (*VHA*) [12], cytochrome c oxidase (*COX*) [10] and heat shock proteins (*HSPs*) [10, 11, 14]. In addition, many genes associated with metabolic processes have also been associated with responses to long-term salinity acclimation [2, 7]. With the advancement of next-generation sequencing (NGS) technology, RNA-seq has become a powerful approach to identify genes and pathways involved in the responses to stress [2, 10, 15, 16]. In the last five years, RNA-seq has been used to study salinity regulation in model and non-model fish species, including spotted sea bass (*Lateolabrax maculatus*) [2], striped catfish (*Pangasianodon hypophthalmus*) [10, 17], Nile tilapia (*Oreochromis niloticus*) [15], and medaka (*Oryzias melastigm*; *Oryzias latipes*) [9].

Whole-genome sequencing of the half-smooth tongue sole has been recently completed, thus providing a valuable reference to unravel the molecular mechanisms underlying salinity adaptation using RNA-seq [18]. In the present study, we conducted an RNA-seq analysis to characterize the salinity-induced changes in gene expression in the liver of half-smooth tongue sole. By assessing the transcriptional variations caused by different salinities, it was possible to identify the genes and the molecular and biological processes involved in salinity adaptation. With these results, investigators can develop functional markers to monitor for contemporary responses to climate change [19] or screen broadly across a species range to predict the potential for adaptation [20]. This information would help to make the fish aquaculture industry successful [2]. Investigating the long-term stress adaptation mechanism also provides insights into understanding plastic and evolutionary responses to various aquatic environments [21–23].

## Methods

### Ethics statement

All animal experiments were conducted in accordance with the guidelines and approval of Institutional Animal Care and Use Committee of Ocean University of China. The field studies did not involve endangered or protected species.

### Salinity challenge experiment and fish sampling

Half-smooth tongue sole were obtained from a local fish farm named shuangying aquatic breeding LLC in Lijin, China. The fish were reared in commercial fish ponds (5 m*5 m*1 m) for 10 months, under controlled conditions (20 ± 0.5 °C; 14:10 h light/dark cycle; O^2^ ≥ 4 ng/ml; 30 ppt) and fed a compound feed. Individuals were randomly divided into six experimental tanks (*N* = 40 in each tank) and acclimatized at an optimal salinity of 30 ppt for a period of 7 days before the start of the salinity trial. For the trials, we only selected female half-smooth tongue of similar lengths, since different patterns of expression have been detected for certain genes between male and female *C. semilaevis* [24–27]. Six experimental tanks were equally divided into two groups including the low salinity group (LS_L group) and high-salinity group (HS_L group, normal living salinity, acted as a control salinity group). Each group had three replicates. The HS_L group was maintained at 30 ppt during the experiment, whereas the salinity in the LS_L group was decreased by 5 ppt per day over a period of three days towards a target of 15 ppt. The experiment lasted for 60 days. During sampling, the experimental fish were anesthetized using 0.1% tricaine methanesulfonate (MS-222), and livers from three female individuals in each tank were dissected, immediately frozen in liquid nitrogen, and stored at − 80 °C until RNA extraction. Each biological replicate represented three pooled female fish livers to make representative samples for deep sequencing analysis. All animal handling was carried out in accordance with the ethical guidelines and protocols of Ocean University of China’s Animal Care Committee.

### RNA sequencing

Total RNA was extracted from livers using RNAiso reagent (TakaRa, Japan) according to the manufacturer’s instruction. Three fish livers from each tank were pooled for RNA extraction. RNA quality was determined using an Agilent Bioanalyzer system (Agilent Technologies, US), and only samples with an RNA Integrity Number (RIN) greater than 7.0 were used for RNA library construction. Six cDNA libraries were prepared from the RNA samples of two groups according to the manufacturer’s recommendation (New England Biolabs, US). The libraries were then sequenced by the Novogene company (China) using the Illumina HiSeq 2500 platform (125 bp, paired-end reads). Raw data (raw reads) in fastq format were first processed through in-house Perl scripts. In this step, the Q20, Q30 and GC content of the clean data were calculated, and the sequencing quality was assessed. At the same time, clean data (clean reads) were obtained by removing the reads containing adaptors, reads with more than 10% poly-N and reads of low quality (the number of bases with sQ < = 5 accounts for more than 50% of the total read length) from the raw data. All downstream analyses were based on high quality clean data.

### Reference genome sequence and read mapping

The clean RNA-seq data were aligned to the reference genome (Accession number: PRJNA251742) using TopHat (version 2.0.9) with mismatch 2 and other parameters set as default. Sequence reads that were mapped to multiple genes or positions were removed. HTSeq v0.6.1 with -m union was used to count the number of reads mapped to each gene. For normalization, the count for each gene was divided by the number of fragments per kilobase of transcript sequence per million base pairs sequenced in each sample (FPKM; fragments per kilobase per million reads) [28].

### Differential expression analysis, gene ontology (GO) and Kyoto encyclopedia of genes and genomes (KEGG) enrichment analysis

After performing a quality check of the raw count data, the differences in gene expression between the LS_L group and HS_L group were analyzed using DESeq2 [29]. Read count data were used as input for the program. Differential expression was tested at an adjusted *P*-value < 0.05 using the Benjamini-Hochberg procedure. Annotation of the DEGs was achieved through BLASTN similarity searches against the whole-genome sequence of half-smooth tongue sole. To characterize the DEGs with Gene Ontology (GO) terms, which have three main categories (biological process, cellular component and molecular function), GO enrichment analysis was conducted using the GOseq R package [30] with correction of gene length bias. Significantly enriched GO terms were ranked using corrected *P*-values less than 0.05. The results provided a broad overview of groups of genes cataloged in the three ontology vocabularies. KOBAS software was used to retrieve the statistical enrichment of differential expression genes in KEGG pathways (http://www.genome.jp/kegg/) [31]. FDR ≤ 0.05 indicated relevant pathways within which regulated genes were significantly enriched.

### Data validation by quantitative real-time PCR (qPCR)

To verify the accuracy of transcriptomic sequencing data, eleven DEGs were selected for quantitative real-time PCR analysis. Total RNA from the liver was extracted individually from the LS_L group and HS_L group. RNA samples were analyzed in biological triplicate and technical triplicate for qPCR. Quantitative PCR was performed using StepOnePlus™ (American) and the SYBR Premix Ex TaqTM (TliRNaseH Plus) Kit (TaKaRa, Japan, Code No. RR420A) according to the manufacturer’s protocol. Gene-specific primers of eleven target genes and one housekeeping gene (*18 s*) [32] were designed using Oligo 6.0 software and synthesized by BGI (The Beijing Genomics Institute). The primer sequences are listed in Additional file 1: Table S1. The 20-μl PCR mixture consisted of 10 μl SYBR®Premix Ex Taq (TliRNaseH Plus), 0.4 μl PCR Forward Primer, 0.4 μl PCR Reverse Primer, 0.4 μl ROX Reference Dye (50×), 2 μl DNA template, and RNase-free water to a total volume of 20 μl. The qPCRs were performed with the following conditions: denaturation at 95 °C for 30 s, 40 cycles of denaturation at 95 °C for 5 s, annealing at 60 °C for 30 s, and extension at 72 °C for 30 s. Relative gene expression was calculated using the 2^-ΔΔCt^ method [33]. Excel software was used to calculate the correlation coefficient between quantitative expression by qPCR and by transcriptome analysis.

### Availability of supporting data

The sequencing data from this study have been submitted to the NCBI Sequence Read Archive (SRA) (https://www.ncbi.nlm.nih.gov/sra) under the accession number SRP071827. The raw and processed data have been submitted to the NCBI Gene Expression Omnibus (GEO) (https://www.ncbi.nlm.nih.gov/gds) under the accession number GSE111312.

## Results

### Illumina sequencing and reads mapping

A total of 342.36 million raw reads were obtained, including 172.19 million reads in the LS_L group and 170.17 million in the HS_L group. The correlation coefficients between replicated samples were greater than 0.88 (Additional file 2: Figure S1). After preprocessing and removal of low-quality sequences, a total of 330.39 million clean reads were obtained, including 166.07 million reads in the LS_L group and 164.32 million in the HS_L group. Ten GB of clean bases were generated for each group. More than 94% of bases had a base accuracy of 99% and more than 89% of bases had a base accuracy of 99.9%. These clean reads were mapped against the annotated genome of the half-smooth tongue sole. A total of 282.10 million reads were successfully mapped. A total of 271.09 million reads were uniquely mapped (Table 1).

**Table 1:** Illumina sequencing and mapping statistics of the liver transcriptomes of half-smooth tongue sole

Sample name	LS_L_1	LS_L_2	LS_L_3	HS_L_1	HS_L_2	HS_L_3
Raw reads (× 10^6^)	55.95	56.01	60.23	54.49	58.77	56.91
Clean reads (× 10^6^)	54.82	54.40	56.85	51.93	57.30	55.09
Clean bases	3.43G	3.4G	3.55G	3.25G	3.58G	3.44G
Q20(%)	95.00	95.35	95.31	94.34	94.72	94.44
Q30(%)	90.67	91.29	91.22	89.48	90.00	89.55
Total mapped (×10^6^)	45.99	46.75	48.92	43.74	49.41	47.29
Mapping rate (%)	83.90%	85.94%	86.04%	84.24%	86.22%	85.84%
Uniquely mapped (×10^6^)	44.39	45.06	47.01	41.99	47.01	45.63
Uniquely mapped rate (%)	80.98%	82.84%	82.70%	80.86%	82.04%	82.82%

### Differential expression analysis between livers from the LS_L and HS_L groups

**Fig. 1:**
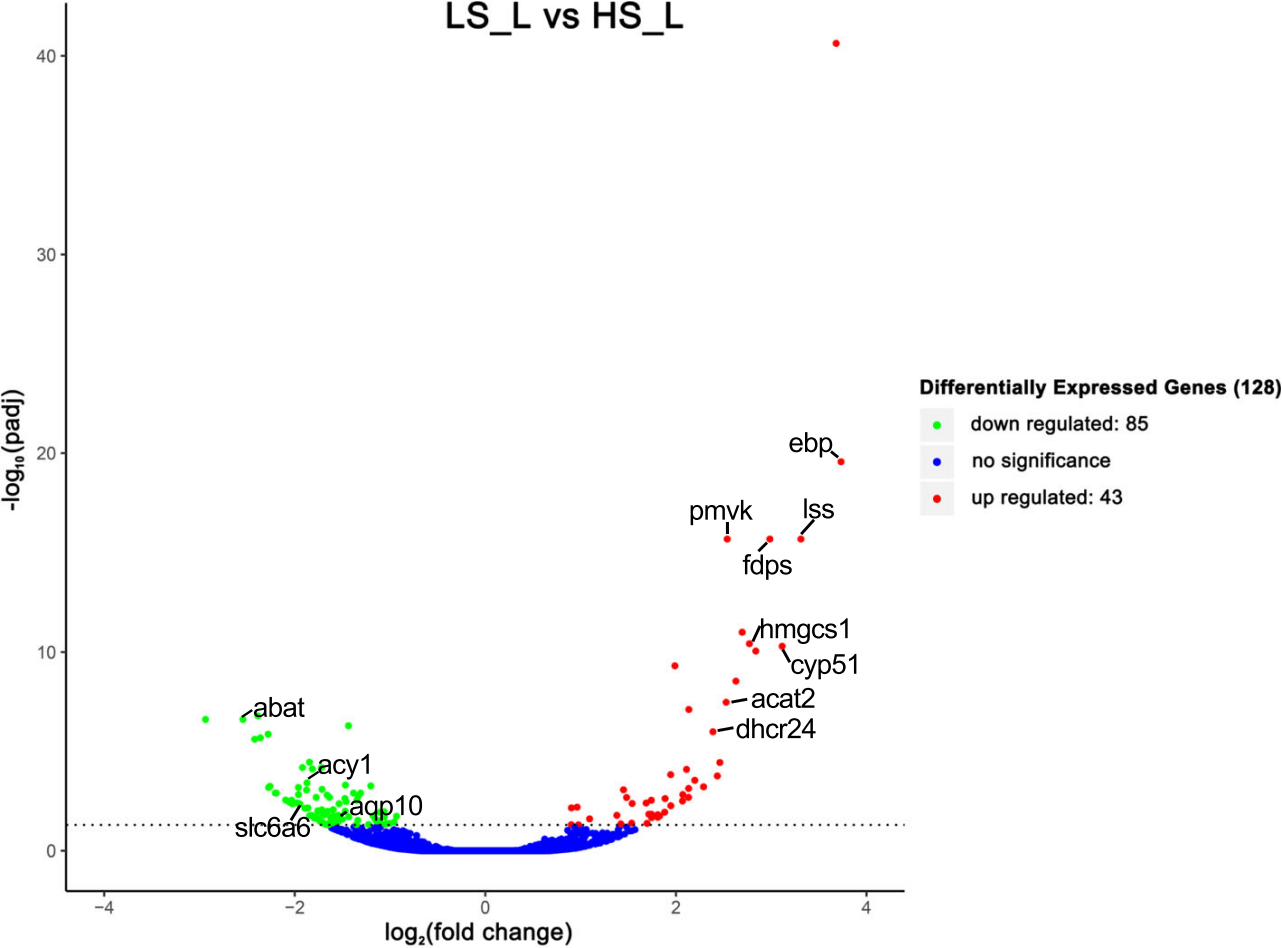
Volcano plot showing the gene expression differences between the LS_L group and HS_L group. Red dots and green dots indicate the significantly up-regulated and down-regulated genes, respectively. Blue dots show genes with no significant differences in expression between the LS_L and HS_L group. The horizontal axis represents the log-ratio (gene expression fold change in different samples) and the vertical axis represents the probability for each gene of being differentially expressed. The total DEG number identified by FDR-adjusted *P*-value < 0.05 is shown

To identify the differentially expressed genes, transcriptome data of half-smooth tongue sole livers from the LS_L group and HS_L group were analyzed. One hundred twenty-eight candidate genes were significantly differentially expressed between the LS_L group and HS_L group using the criteria of FDR-adjusted *P*-value< 0.05 and |Log_2_(fold change)| > 1. Compared with the HS_L group, 43 DEGs were up-regulated and 85 DEGs were down-regulated in the LS_L group (Fig. 1). Detailed DEG information is shown in Additional file 3: Table S2. To illustrate the differential expression of genes detected in the liver among different salinities, a heat map of FPKM-normalized transcript counts for the DEGs in each pairwise comparison was generated (Additional file 4: Figure S2).

### Gene ontology analysis

**Fig. 2:**
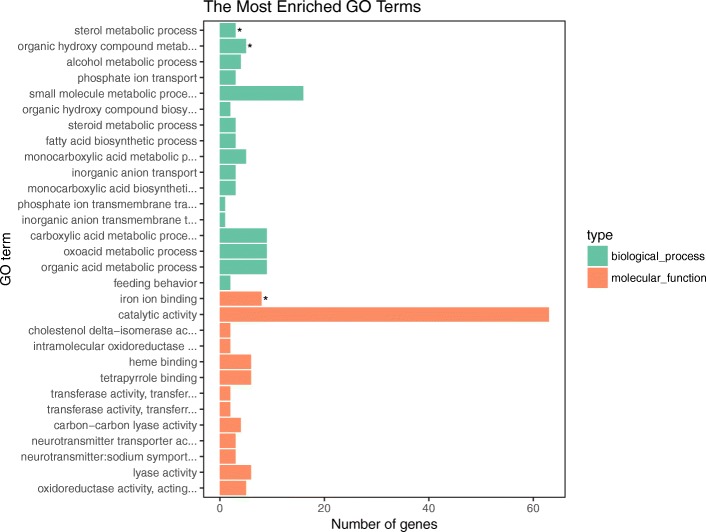
Gene Ontology (GO) terms based on all DEGs of the half-smooth tongue sole during acclimation to low salinity (* indicates significantly enriched GO terms)

All annotated genes were divided into three categories: molecular function, cellular component and biological process. Of the 34,751 unigenes, 27,828 were assigned to 881 GO categories (Additional file 5: Table S3). GO enrichment analysis of the DEGs showed the top processes according to the number of genes and the enrichment level (*P*-value < 0.05). According to biological process, *sterol metabolic process* (GO: 0016125) and *organic hydroxy compound metabolic process* (GO: 1901615) were highly ranked in GO enrichment. According to molecular function, *iron ion binding* (GO: 0005506) was markedly enriched. There was no significant enrichment of GO terms in the cellular component category (Fig. 2). More GO terms were identified based on the up-regulated genes. These terms included *sterol metabolic process* (GO: 0016125), *alcohol metabolic process* (GO: 0006066), *organic hydroxy compound metabolic process* (GO: 1901615), *steroid metabolic process* (GO: 0008202) and *fatty acid biosynthetic process* (GO: 0006633), which were all significantly enriched in the biological process category. In the molecular function category, two more significant enriched GO terms were found, namely *cholesterol delta-isomerase activity* (GO: 0047750) and *intramolecular oxidoreductase activity* (GO: 0016863) (Additional file 6: Figure S3).

### KEGG analysis

**Fig. 3:**
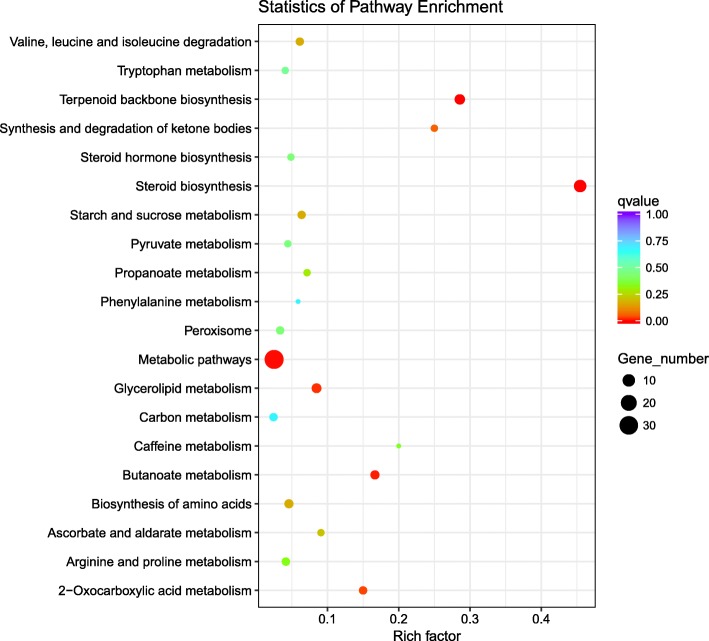
Scatter plot showing KEGG pathway enrichment among the DEGs. The vertical axis represents the pathway categories, and the horizontal axis shows the enrichment factor. The point size shows the number of DEGs among the pathway. The bigger the point size, the more genes in the pathway. The point color shows different Q values as indicated on the right. Metabolic pathways contained the most DEGs

KEGG pathway analysis identifies molecular interaction networks within cells and helps to elucidate the potential biological functions of analyzed genes. As shown in Figs. 3, 6 pathways were found to be highly enriched by KEGG analysis. In which, metabolic pathways were the most frequently represented pathways in response to long-term hypotonic stress. The DEGs enriched in these pathways are shown in Table 2. The metabolic pathways contained 32 DEGs, whereby 15 of these genes were up-regulated in low salinity conditions, and 17 were down-regulated during acclimation to low salinity. Some pathways shared the same DEGs. Genes highlighted in the steroid biosynthesis pathway, terpenoid backbone biosynthesis pathway, glycerolipid metabolism pathway, and 2-oxocarboxylic acid metabolism pathway also functioned in the metabolic pathways. In the butanoate metabolism pathway, all of the highlighted genes except for the *aacs* gene were also involved in metabolic pathways.

**Table 2:** The most enriched KEGG pathways from *C. semilaevis* in response to long-term hypotonic stress

Pathways	Corrected *P*-Value	DEGs
Steroid biosynthesis	1.86E-10	*sc5d*, *cyp51*, *ebp*, *LOC103395808*, *LOC103389307*↓, *LOC103389284*↓, *lss*, *msmo1*, *dhcr7*, *dhcr24*
Terpenoid backbone biosynthesis	2.43E-05	*acat2*, *pmvk*, *hmgcs1*, *fdps*, *mvk*, *hmgcr*
Metabolic pathways	0.001746903	*LOC103388276*↓, *LOC103392251*↓, *LOC103397733*, *sc5d*, *acat2*, *cyp51*, *abat*↓, *LOC103399583*↓, *LOC103381542*↓, *ebp*, *LOC103387723*↓, *LOC103395808*, *gamt*↓, *ddc*↓, *acy1*↓, *LOC103378976*↓, *pmvk*, *fdps*, *LOC103396493*↓, *hmgcs1*, *LOC103390352*↓, *mvk*, *LOC103389307*↓, *idh1*, *LOC103389284*↓, *hmgcr*, *lss*, *LOC103396997*, *msmo1*, *dhcr7*, *dhcr24*, *LOC103377813*↓
Butanoate metabolism	0.005924384	*aacs*, *acat2*, *abat*↓, *hmgcs1*
Glycerolipid metabolism	0.014735483	*LOC103387723*↓, *LOC103397733*, *LOC103389307*↓, *LOC103389284*↓, *LOC103396997*
2-Oxocarboxylic acid metabolism	0.030077568	*LOC103399583*↓, *acy1*↓, *idh1*

↓ indicates that the gene was down-regulated.

### The classification of gene function analysis

**Fig. 4:**
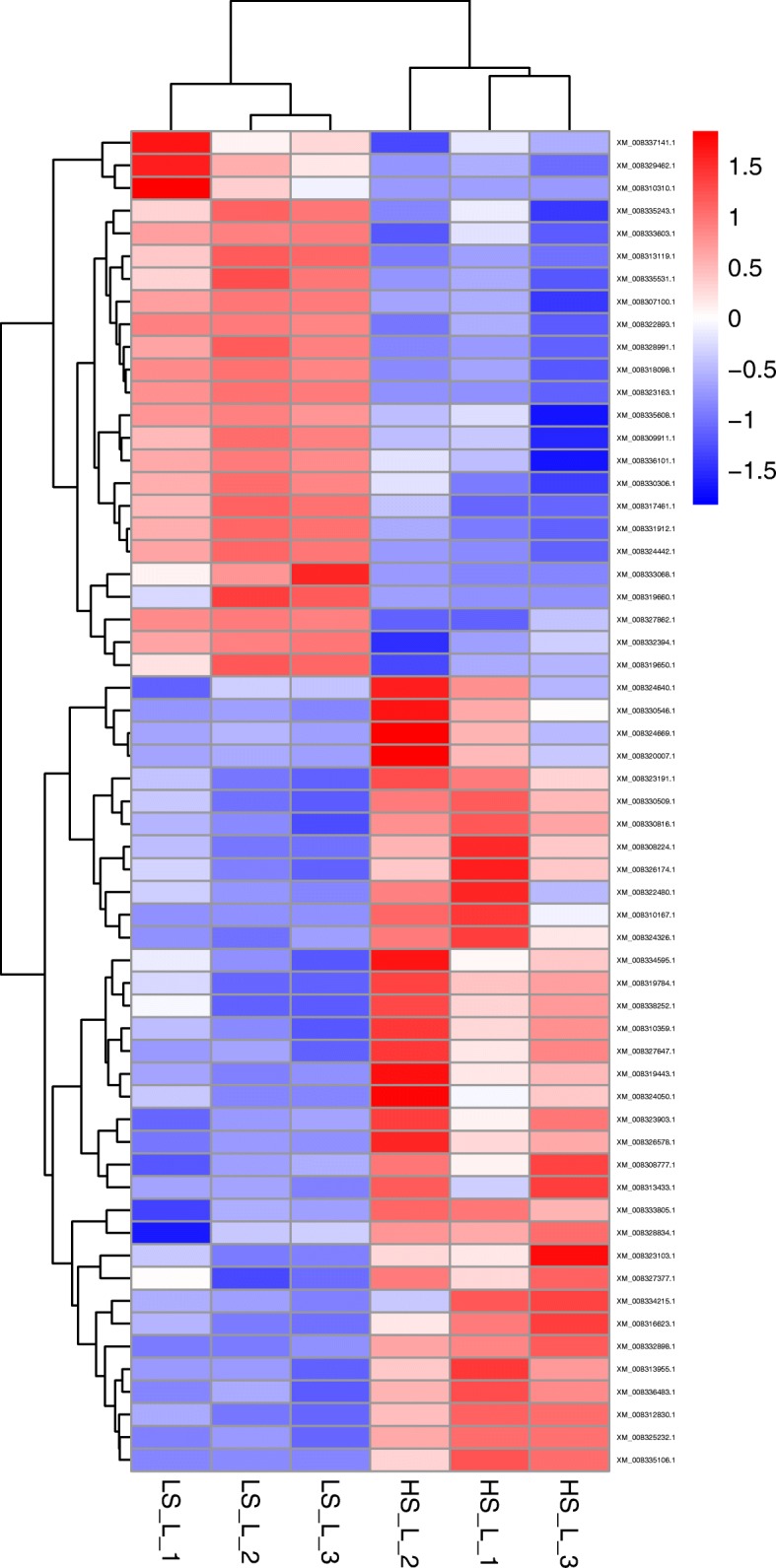
Heat map analysis of DEGs function clustering in the liver from the LS_L group and HS_L group

Based on the combination of enrichment analysis, annotation and manual literature searches, gene function annotation resources showed various candidate DEGs potentially associated with salinity adaptation and osmoregulation. These DEGs are involved in metabolism, osmoregulation and ion transport, signal transduction, immune response and stress response, and cytoskeleton remodeling (Table 3). To illustrate the differential expression of genes detected in the liver among different salinities, a heat map of FPKM-normalized transcript counts for the DEGs in two groups was generated (Fig. 4).

**Table 3:** List of functional groups and related liver DEGs from *C. semilaevis* in response to long-term hypotonic stress

Function group	Gene name	Gene ID	Gene function	Log_2_(fold change)
Metabolism				
*ebp*		XM_008318098.1	emopamil binding protein (sterol isomerase)	+ 3.73
*lss*		XM_008328991.1	lanosterol synthase (2%2C3-oxidosqualene-lanosterol cyclase)	+ 3.31
*cyp51*		XM_008331912.1	lanosterol 14-alpha demethylase	+ 3.12
*fdps*		XM_008323163.1	farnesyl diphosphate synthase	+ 2.99
*hmgcs1*		XM_008324442.1	hydroxymethylglutaryl-CoA synthase%2C cytoplasmic-like	+ 2.77
*pmvk*		XM_008322893.1	phosphomevalonate kinase	+ 2.54
*acat2*		XM_008313119.1	acetyl-CoA acetyltransferase 2	+ 2.53
*dhcr24*		XM_008307100.1	24-dehydrocholesterol reductase	+ 2.39
*msmo1*		XM_008317461.1	methylsterol monooxygenase 1	+ 2.11
*dhcr7*		XM_008309911.1	7-dehydrocholesterol reductase	+ 1.89
*sc5d*		XM_008333068.1	sterol-C5-desaturase%2C transcript variant X1	+ 1.83
*LOC103396997*		XM_008335243.1	endothelial lipase-like	+ 1.74
*hmgcr*		XM_008335608.1	3-hydroxy-3-methylglutaryl-CoA reductase	+ 1.74
*mvk*		XM_008335531.1	mevalonate kinase	+ 1.54
*LOC103397733*		XM_008336101.1	endothelial lipase-like	+ 1.54
*idh1*		XM_008327862.1	isocitrate dehydrogenase 1 (NADP+)%2C soluble	+ 1.48
*LOC103395808*		XM_008333603.1	3-keto-steroid reductase-like	+ 1.45
*abat*		XM_008330509.1	4-aminobutyrate aminotransferase	−2.54
*acy1*		XM_008319784.1	aminoacylase 1	−1.87
*ddc*		XM_008323903.1	dopa decarboxylase (aromatic L-amino acid decarboxylase)	− 1.82
*LOC103377813*		XM_008308777.1	alkaline phosphatase-like	−1.79
*LOC103389307*		XM_008324669.1	bile salt-activated lipase-like	−1.73
*LOC103388276*		XM_008323191.1	pyruvate kinase PKM-like	−1.72
*LOC103389284*		XM_008324640.1	bile salt-activated lipase-like	−1.69
*LOC103378976*		XM_008310359.1	phospholipase D1-like	−1.66
*gamt*		XM_008333805.1	guanidinoacetate N-methyltransferase	−1.64
*LOC103387723*		XM_008322480.1	inactive pancreatic lipase-related protein 1-like	−1.62
*LOC103399583*		XM_008338252.1	aminoacylase-1-like	−1.61
*LOC103392251*		XM_008328834.1	uricase-like	−1.58
*LOC103396493*		XM_008334595.1	pancreatic alpha-amylase-like	−1.54
*LOC103390352*		XM_008326174.1	UDP-glucuronosyltransferase 2A1-like%2C transcript variant X3	−1.31
*LOC103381542*		XM_008313955.1	neutral alpha-glucosidase C-like	−1.20
Osmoregulation and ion transport				
*LOC103392748*		XM_008329462.1	chromodomain-helicase-DNA-binding protein 1-like	+ 1.95
*LOC103394918*		XM_008332394.1	sodium-dependent dopamine transporter-like	+ 1.38
*slc6a6*		XM_008319443.1	solute carrier family 6 (neurotransmitter transporter)%2C member 6%2C transcript variant X1	−1.95
*rem2*		XM_008334215.1	RAS (RAD and GEM)-like GTP binding 2	−1.65
*aqp10*		XM_008323103.1	aquaporin 10	−1.58
*LOC103391201*		XM_008327377.1	ependymin-like	−1.43
*ankh*		XM_008308224.1	ANKH inorganic pyrophosphate transport regulator	−1.33
*LOC103389703*		XM_008325232.1	sodium- and chloride-dependent GABA transporter 2-like	−1.06
Signal transduction				
*LOC103385696*		XM_008319660.1	taste receptor type 1 member 3	+ 2.43
*ghsr*		XM_008310310.1	growth hormone secretagogue receptor	+ 2.29
*hmgcr*		XM_008335608.1	3-hydroxy-3-methylglutaryl-CoA reductase	+ 1.74
*LOC103398491*		XM_008337141.1	cocaine- and amphetamine-regulated transcript protein-like	+ 1.72
*LOC103395241*		XM_008332898.1	olfactory receptor 24-like	−2.94
*abat*		XM_008330509.1	4-aminobutyrate aminotransferase	−2.54
*LOC103390650*		XM_008326578.1	ankyrin repeat and SOCS box protein 5-like	−2.03
*LOC103393547*		XM_008330546.1	TANK-binding kinase 1-binding protein 1-like	−1.86
*LOC103383485*		XM_008316623.1	ATP-binding cassette sub-family G member 2-like	−1.81
*LOC103378821*		XM_008310167.1	galanin receptor type 1-like	−1.78
*LOC103381198*		XM_008313433.1	scavenger receptor class A member 3-like	−1.75
*rem2*		XM_008334215.1	RAS (RAD and GEM)-like GTP binding 2	−1.65
*LOC103388851*		XM_008324050.1	C-X-C chemokine receptor type 3-like	−1.64
*dpf3*		XM_008312830.1	D4%2C zinc and double PHD fingers%2C family 3%2C transcript variant X3	−1.60
*LOC103392251*		XM_008328834.1	uricase-like	−1.58
*LOC103396858*		XM_008335106.1	zinc finger protein 79-like	−1.54
*npr1*		XM_008330816.1	natriuretic peptide receptor 1	−1.44
*LOC103391201*		XM_008327377.1	ependymin-like	−1.43
*LOC103398008*		XM_008336483.1	phosphatidylinositol 3-kinase regulatory subunit alpha-like%2C transcript variant X1	−1.35
Immune response and stress response				
*ebp*		XM_008318098.1	emopamil binding protein (sterol isomerase)	+ 3.73
*LOC103385685*		XM_008319650.1	coiled-coil domain-containing protein 114-like	+ 2.20
*LOC103398491*		XM_008337141.1	cocaine- and amphetamine-regulated transcript protein-like	+ 1.72
*endou*		XM_008320007.1	endonuclease%2C polyU-specific	−2.03
*LOC103393547*		XM_008330546.1	TANK-binding kinase 1-binding protein 1-like	−1.86
*LOC103381198*		XM_008313433.1	scavenger receptor class A member 3-like	−1.75
*LOC103388276*		XM_008323191.1	pyruvate kinase PKM-like	−1.72
*fam163b*		XM_008324326.1	family with sequence similarity 163%2C member B	−1.54
Cytoskeleton remodeling				
*srebf2*		XM_008330306.1	sterol regulatory element binding transcription factor 2	+ 1.42
*ablim3*		XM_008327647.1	actin binding LIM protein family%2C member 3	−1.88
*LOC103393547*		XM_008330546.1	TANK-binding kinase 1-binding protein 1-like	−1.86
*LOC103381198*		XM_008313433.1	scavenger receptor class A member 3-like	−1.75
*LOC103398008*		XM_008336483.1	phosphatidylinositol 3-kinase regulatory subunit alpha-like%2C transcript variant X1	−1.35

### Validation of RNA-seq results by qPCR

**Fig. 5:**
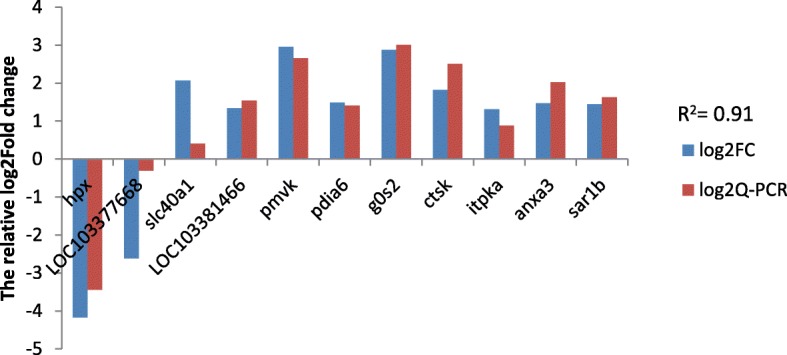
Validation by qPCR. Comparison of the relative log_2_(fold changes) between RNA-seq and qPCR after salinity acclimation compared to the control, as normalized to expression of the *18 s* gene

To verify the accuracy of the RNA-seq data, eleven genes were chosen for validation by qPCR. These genes included *hpx*, *LOC103377668*, *slc40a1*, *LOC103381466*, *pmvk*, *pdia6*, *g0 s2*, *ctsk*, *itpka*, *anxa3* and *sar1b*. As shown in Fig. 5, the correlation coefficient of the relative log_2_(fold changes) was 0.91 between RNA-seq and qPCR after salinity acclimation, suggesting the correctness of the bioinformatics analysis for the transcriptomic sequencing data.

## Discussion

With the advancement of transcriptome sequencing, RNA-Seq has emerged as a powerful tool to study the molecular response in fish impacted by environmental changes [23]. In the present study, we conducted a transcriptome analysis to reveal the changes of gene expression profiles in the liver of half-smooth tongue sole when responding to salinity changes. A total of 128 DEGs were identified during acclimation to low salinity conditions. These DEGs are enriched in several biological processes, including lipid metabolism, steroid biosynthesis, osmoregulation and ion transport, and signal transduction. Data from our studies are consistent with previous findings that alteration of liver metabolism plays a critical role in salinity adaptation in *L. maculatus* [2]. This is probably due to the fact that additional energy is needed for ion transport during acclimation to different osmotic environments in euryhaline fishes, and liver is the main source of energy for osmoregulatory organs to help with the synthesis and function of enzymes and transporters [8].

### Metabolism was one of the most enriched pathways during acclimation to a low salinity environment

It has been reported that euryhaline fish can utilize lipids as an energy source when encountering osmotic stress [9, 21, 34]. In our study, we found that 32 genes in the metabolic pathways were identified, in which genes *ebp*, *cyp51*, *fdps*, *pmvk*, *hmgcs1*, *lss*, *dhcr24*, *acat2*, *dhcr7*, and *abat* were involved in lipid metabolism under the low salinity environment. The *cyp51* gene encodes lanosterol 14-alpha demethylase, a key enzyme involved in lipid metabolism and steroid biosynthesis [35, 36]. Interestingly, the expression of *cyp51-like* gene was down-regulated in *Oreochromis mossambicus*, a euryhaline freshwater fish, but up-regulated in *O. niloticus*, a stenohaline freshwater fish when responding to increased salinity environment. This indicated the different role of *cyp51-like* in different species during acclimation to different salinity conditions [37]. It has been shown that reduced expression of *cyp51* gene might contribute to the decreased lipid levels in both XBP1- and IRE1α-deficient mice, whose lipid metabolism were significantly down-regulated [35]. In our study, we found that cyp51 was up-regulated during acclimation to low salinity conditions, indicating the increased lipid metabolism and lipid levels in *C. semilaevis*. The novel liver protein acetyl-CoA acetyltransferase-2 (ACAT2) is involved in beta-oxidation and lipid metabolism [38]. In *L. maculatus*, *acetyl-CoA acetyltransferase* (*acat*) was down-regulated during adaptation to high salinity conditions [2]. The down-regulation of expression of *acat1* suggested that the amount of energy produced from the TCA cycle might be low in FW milkfish under hypothermia [39]. In our study, the expression of *acat2* was up-regulated when salinity was decreased, suggesting that the amount of energy might be high.

Terpenoids are the most abundant lipids and precursors in the synthesis of complex secondary metabolites such as sterols and steroids [40]. Owing to the increasing amount of energy, the demand for terpenoids is highly urgent. The terpenoid backbone biosynthesis pathway was first found to be involved in salinity adaptation. The production of terpenoids requires six key enzymes and the expression of these genes (*acat2*, *hmgcs1*, *hmgcr*, *mvk*, *pmvk* and *fdps*) were shown to be up-regulated in our study. ACAT2, HMGCS1, HMGCR, MVK, PMVK are all crucial enzymes involved in mevalonate pathway, which is an important process of terpenoid backbone biosynthesis and is considered to be the only pathway of producing the precursors isopentenyl-PP (IPP) and dimethylallyl-PP (DMAPP) [41]. Next, the transformation of DMAPP and IPP was catalyzed into farnesyl-PP (FPP), which finally produces terpenoids with the help of farnesyl diphosphate synthase (FDPS) [42, 43]. These highly activated enzymes involved in lipid metabolism and terpenoids biosynthesis indicated the increased amount of energy and lipids in the present study. It can be deduced that lipid metabolism and biosynthesis were more likely needed during hypotonic acclimation.

Steroid biosynthesis played a pivotal role in response to salinity stress in aquatic animals [15]. Our study identified some biosynthesis-related genes such as *ebp*, *lss*, *dhcr7*, *dhcr24*, and *cyp51*, and these genes also played roles in metabolic pathways. Sterols are important compounds in many biological membranes; they not only act as cell membrane components, but also possess transport capability [44, 45]. Emopamil binding protein (ebp), also known as sterol isomerase, is an essential enzyme in the sterol biosynthesis pathway in eukaryotes [46]. In the present study, the *ebp* gene expression was up-regulated to active sterol biosynthesis in response to salinity stress, indicating an increase in sterol levels in the liver. Lanosterol is an upstream precursor of sterol biosynthesis for all animal and fungal steroids (especially cholesterol) [47]. The *lss* gene encodes lanosterol synthase and a null mutation for *lss* decreased cholesterol levels in the Shumiya cataract rat [48]. Increased *lss* expression in our case may indicate improved cholesterol levels in the liver. After the synthesis of lanosterol, the production of 14-demethylation of lanosterol catalyzed by CYP51 eventually yields cholesterol after a series of complicated reactions. In our study, *cyp51* was up-regulated during acclimation to low salinity conditions, indicating active cholesterol synthesis in *C. semilaevis*. In a previous study of constant salinity change, *lss* and *dhcr24* in the steroid biosynthesis pathway were found to be up-regulated in Nile tilapia (*O. niloticus*) [15], which is consistent with the data from our present study.

Cholesterol biosynthesis is crucial in the relationship between osmoregulation and steroid because the cholesterol induced by salinity fluctuation is related to the physical properties of cell membranes [15, 49]. An increasing trend was found for high-density lipoprotein cholesterol as salinity decreased in *Eriocheir sinensis* [50]. Genes *24-dehydrocholesterol reductase* (*dhcr24*) and *7-dehydrocholesterol reductase* (*dhcr7*) are the final enzymes required for cholesterol biosynthesis [51]. A stronger evolutionary correlation was found between DHCR24 and Na^+^,K^+^-ATPase than between DHCR24 and any other membrane protein investigated, indicating that cholesterol evolved together with Na^+^,K^+^-ATPase in multicellular animals to support Na^+^,K^+^-ATPase activity [52]. Previous studies suggested that the cholesterol production induced by salinity might influence the physical properties of cell membranes [49]. Transcriptome profiling in *O. niloticuspivotal* showed the vital roles of sterol metabolism in response to salinity stress [15]. Hyposmotic stress may cause damage to cell membranes. Given that five up-regulated DEGs in our study contributed to cholesterol synthesis, we can infer that biosynthesis of cholesterol played an important role in the relationship between osmoregulation and steroid production in order to maintain osmotic homeostasis in a long-term low salinity environment. Cholesterol function was more likely related to the physical properties of cell membranes. In addition, cholesterol is the precursor of cortisol, a hormone which responds to various stress conditions and can be widely used as a biomarker in aquaculture. Previous study has comfirmed that the levels of cortisol were elevated under stress to maintain the homeostasis in fish [53].

It has been reported that the propanoate metabolism pathway was activated in the gill of the pacific white shrimp *Litopenaeus vannamei* when responding to acute low salinity stress [54]. Butanoate metabolism was identified as a new pathway for long-term low salinity responses in our study. The *4-aminobutyrate aminotransferase* (*abat*) gene, also known as *GABA transaminase*, was down-regulated in the metabolic pathways and butanoate metabolism pathway. In the plant *Arabidopsis*, GABA transaminase (GABA-T), the first step of GABA catabolism, was identified as the most responsive to NaCl levels [55]. This is the first report of *abat* in teleosts, and further research is needed to reveal the underlying regulation mechanism.

### Osmoregulation and ion transport were involved in acclimation to long-term low salinity conditions

Osmoregulation is the active regulation of osmotic pressure by means of maintaining a balance of intracellular solute concentration when adapting to the surrounding conditions. Aquaporins (AQPs) represent a family of transmembrane channel proteins that allow osmotic-driven transport of water and small solutes across biological membranes and are found in all living organisms [56]. Our studies here showed that *aquaporin 10* (*aqp10*) was down-regulated in low salinity water*.* Aquaporin 10 is an aquaglyceroporin that is located in the membrane of the mammalian small intestine, where it is believed to participate in transport of both water and solutes [57]. Previous studies have demonstrated that other members of the AQP family play a role in osmoregulation. *AQP1* was often used as a reference gene because it was identified to change in teleosts after salinity challenge [58]. A decrease in AQP-3 can help prevent loss of glycerol from the cell [59]. The previous study also showed a decrease in AQP-8a and AQP-10b expression abundance in the intestine of *Pangasianodon hypophthalmus* when salinity levels increased [17]. Collecitvely, these provided a pivotal hint to clarify water transport activity in teleosts.

The transport of ions across the plasma membrane by transporters is essential to osmoregulation. During accumulation to different salinities, cells can depend on transporters and accumulate intracellular osmolytes, including taurine, betaine, sorbitol and myo-inositol, to balance any osmotic change [60, 61]. The sodium/chloride-dependent taurine transporter (SLC6A6; TauT) has specificity for taurine [62]. Functional analysis of *slc6a6* in pacific oyster *Crassostrea gigas* revealed that *slc6a6* required higher NaCl concentration [62]. In our study, half-smooth tongue sole *slc6a6* mRNA expression was down-regulated under hyposmotic stress. Taurine transporter mRNA has also been found to be highly expressed in the gill of seawater-acclimated Japanese eel (*Anguilla japonica*) [63]. It is speculated that the down-regulation of *slc6a6* in response to hyposmotic stress was induced by a substantial decrease in tissue taurine content following the decrease in internal osmolality.

### Signal transduction was indispensable during acclimation to salinity changes

The initial basis for euryhaline fish to adapt to salinity stress is the efficient mechanisms of osmosensing and osmotic stress signaling [64]. They are the upstream mechanisms that regulate effector protein expression and orchestrate adaptive responses [65]. Signal transduction is activated by ligand-receptor binding and then is propagated through several transducer proteins via phosphorylation or dephosphorylation events [65]. In our results, several genes involved in signal transduction were found differentially expressed between the LS_L group and HS_L group, such as *abat*, *rem2*, *dpf3* and *npr1*. Natriuretic peptides play a central role in fish osmoregulation (especially in seawater). Natriuretic peptides inhibited salt loading, rather than stimulated salt loss in marine fish [66]. The gene expression of its receptor NPR1 was found down-regulated in the present result, indicating the potential role of *npr1* in the signal transduction. These genes found in our study may have important functions in osmo-regulated signaling pathways, which can be used to regulate effector protein expression and orchestrate adaptive responses.

In the present study, we only analyzed coding RNA (mRNA), but not the non-coding RNAs (e.g., lncRNA or microRNA). Future research will be carried out to obtain a comprehensive understanding of the whole transcriptomic regulatory mechanism in salinity adaptation including lncRNA, microRNA and circRNA sequencing.

## Conclusion

The results of this study highlight the response of the liver transcriptome to long-term environmental salinity changes using next-generation sequencing technology. Data from this study indicate that the liver plays a critical role in the long-term salinity adaptation of aquatic animals. A list of candidate genes with differential expression patterns were identified in response to *C. semilaevis* salinity tolerance. These genes were enriched in diverse biological processes including metabolism, osmoregulation and ion transport, signal transduction, immune response and stress response, and cytoskeleton remodeling. Lipid metabolism and biosynthesis were the most remarkable affected genetic pathways during acclimation to low salinity. Some genes such as *abat*, *fdps*, *hmgcs1*, *mvk and pmvk* and *dhcr7* involved in these processes were first reported to be associated with salinity adaptation. This study not only offered a number of candidate genes for salinity adaptation in half-smooth tongue sole but will also facilitate research into extensive transcriptional plasticity to identify underlying physiological adaptations to different salinities for other euryhaline teleosts.

## Additional files

Additional file 1: **Table S1.** Primers used in qPCR validation. (XLSX 11 kb)

Additional file 2: **Figure S1.** Pearson’s correlation coefficients among the LS_L group and HS_L group. (PDF 5 kb)

Additional file 3: **Table S2.** List of DEGs from *C. semilaevis* in response to long-term hypotonic stress. (XLS 50 kb)

Additional file 4: **Figure S2.** Heat map analysis of all DEGs in the liver from the LS_L group and HS_L group. (PDF 13 kb)

Additional file 5: **Table S3.** List of Gene Ontology (GO) enrichment terms in the liver of half-smooth tongue sole. (XLS 221 kb)

Additional file 6: **Figure S3.** Gene Ontology (GO) terms based on up-regulated DEGs of half-smooth tongue sole during acclimation to low salinity (* indicates significantly enriched GO terms). (PDF 5 kb)

## Abbreviations

*C. semilaevis*: *Cynoglossus semilaevis*
COX: Cytochrome c oxidase
DEGs: Differentially expressed genes
DMAPP: Dimethylallyl pyrophosphate
FPKM: Fragments per kilobase per million reads
FPP: Farnesyl pyrophosphate
GO: Gene Ontology
HSPs: Heat shock proteins
IPP: Isopentenyl pyrophosphate
KEGG: Kyoto Encyclopedia of Genes and Genomes
NKA: Na^+^/K^+^-ATPase
Ppt: Parts per thousand
RIN: RNA Integrity Number
VHA: Vacuolar-type H^+^-ATPase
