# Supplementary figures and images for: Liver transcriptome analysis reveals extensive transcriptional plasticity during acclimation to low salinity in Cynoglossus semilaevis
Source: BMC Genomics. 2018 Jun 18;19:464. doi: 10.1186/s12864-018-4825-4 (PMC6006554; doi:10.1186/s12864-018-4825-4)

Pearson correlation between samples

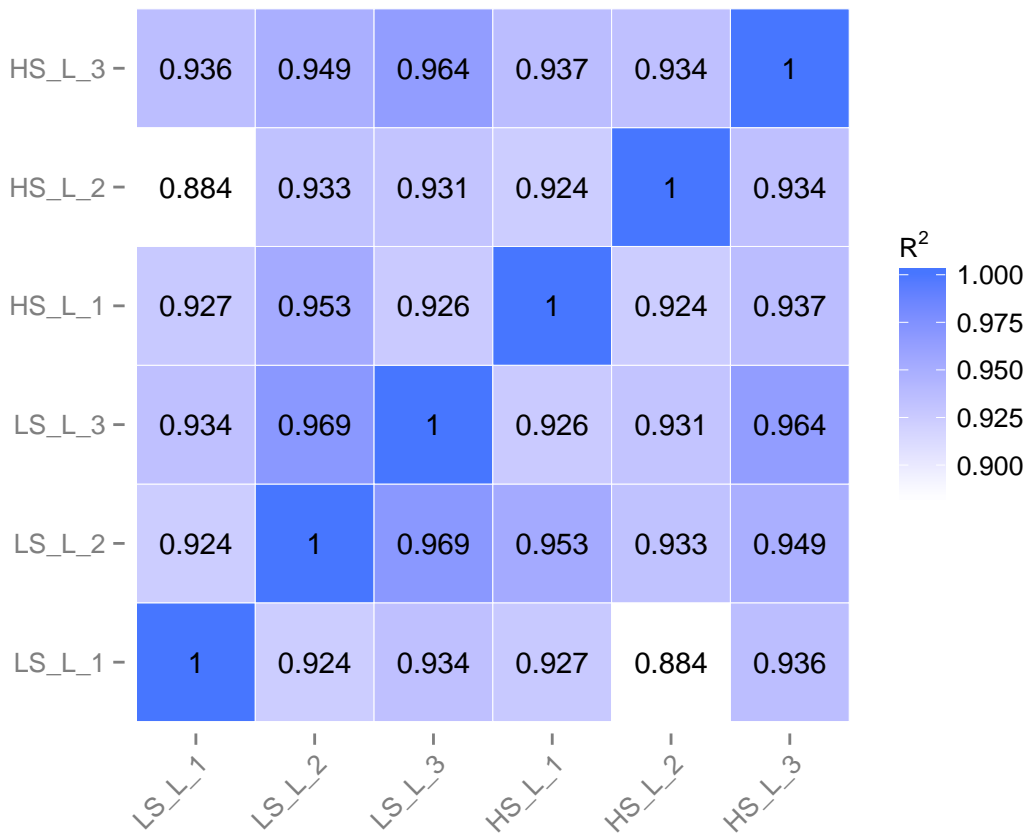

Supplement: Supplementary file 2 — Figure S1. Pearson’s correlation coefficients among the LS_L group and HS_L group. (PDF 5 kb) [file 12864_2018_4825_MOESM2_ESM.pdf]

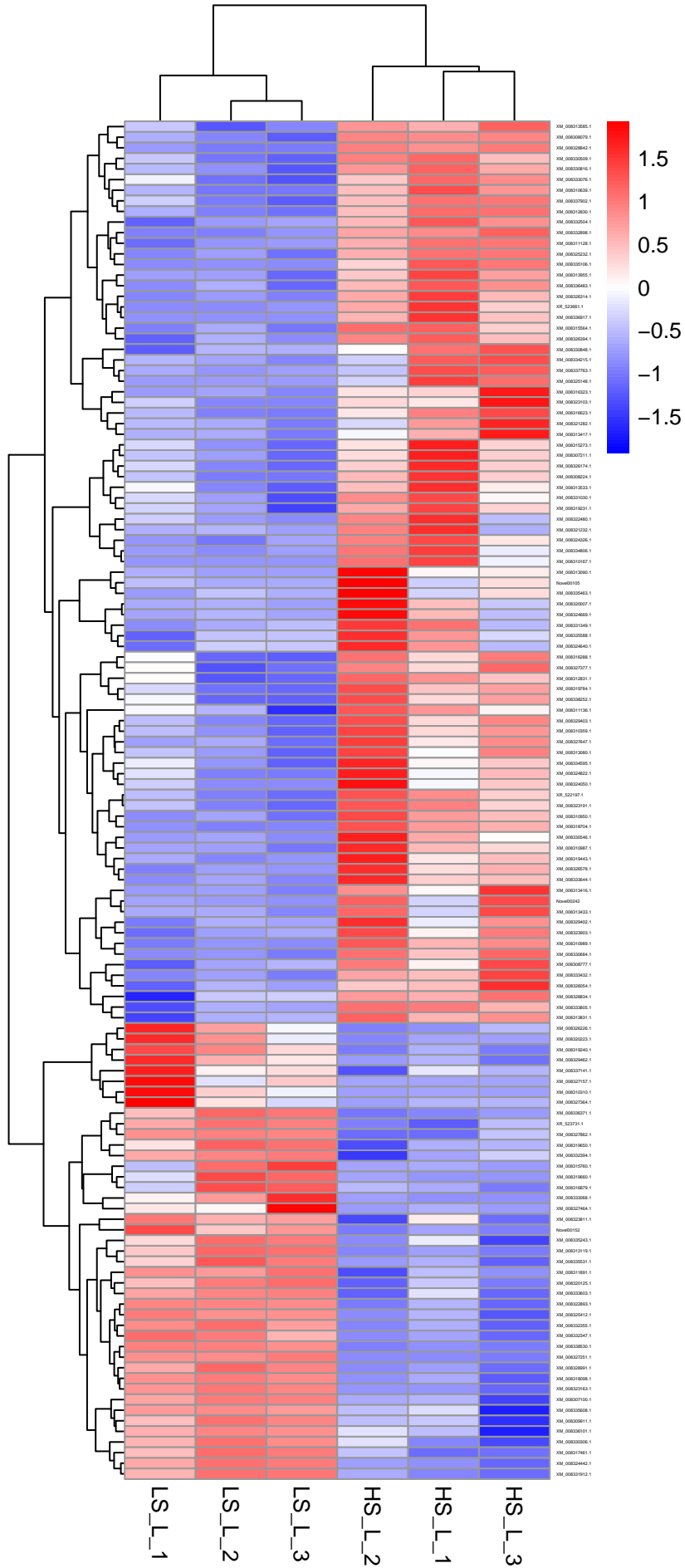

Supplement: Supplementary file 4 — Figure S2. Heat map analysis of all DEGs in the liver from the LS_L group and HS_L group. (PDF 13 kb) [file 12864_2018_4825_MOESM4_ESM.pdf]

# The Most Enriched GO Terms

GO term

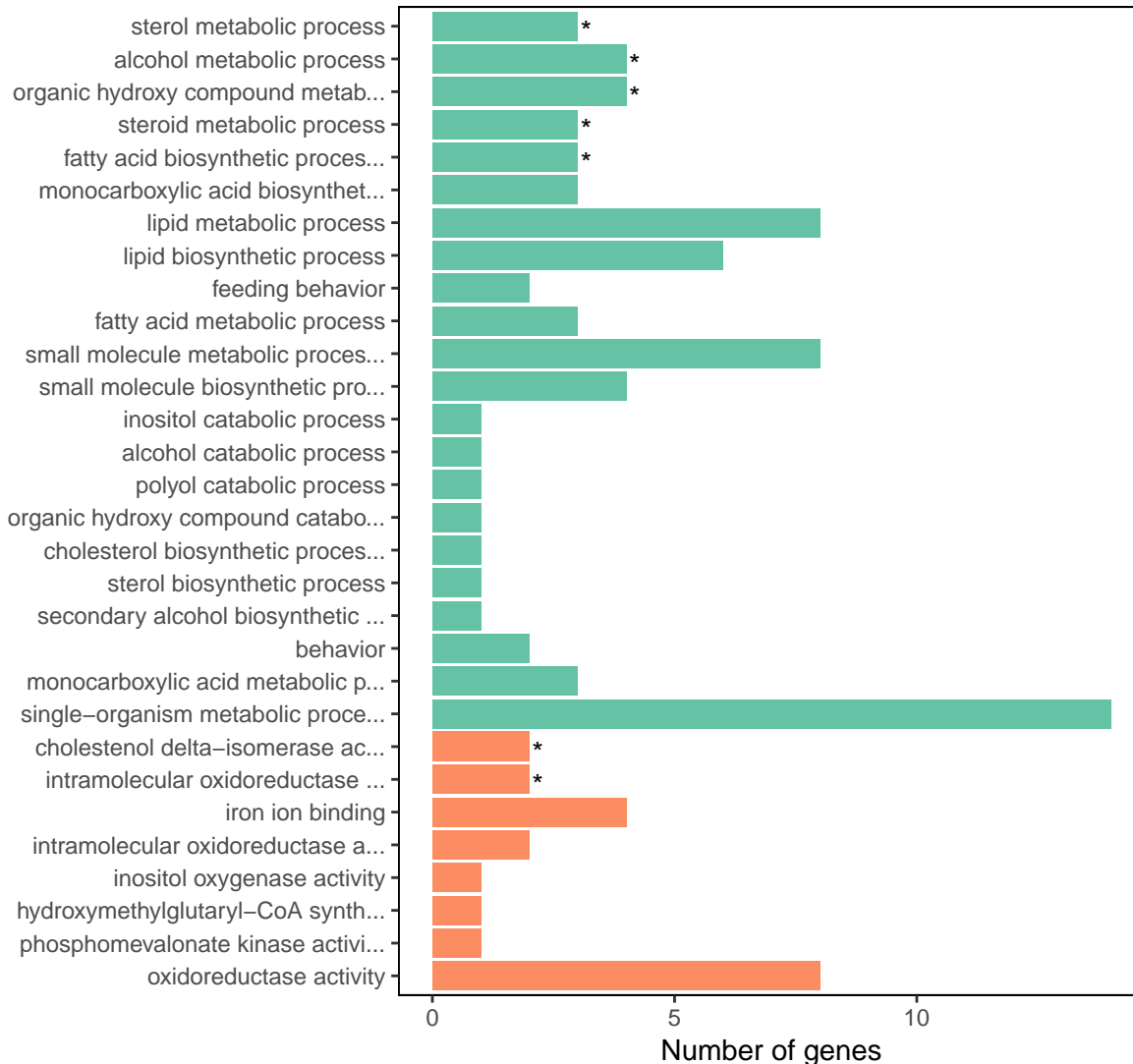

type

biological\_process  
molecular\_function

Supplement: Supplementary file 6 — Figure S3. Gene Ontology (GO) terms based on up-regulated DEGs of half-smooth tongue sole during acclimation to low salinity (* indicates significantly enriched GO terms). (PDF 5 kb) [file 12864_2018_4825_MOESM6_ESM.pdf]
